# Local Perineal Capillary Perforator Flaps: A Minimally Invasive Technique for the Correction of Vulvar Stenosis

**DOI:** 10.3390/jpm14060617

**Published:** 2024-06-08

**Authors:** Anna Amelia Caretto, Giorgia Garganese, Simona Maria Fragomeni, Luca Tagliaferri, Bruno Fionda, Giovanni Scambia, Stefano Gentileschi

**Affiliations:** 1 Unità di Chirurgia Plastica, Dipartimento Scienze Della Salute Della Donna E Del Bambino E Di Sanità Pubblica, Fondazione Policlinico Universitario A. Gemelli IRCCS, Largo Agostino Gemelli 8, 00168 Roma, Italy; 2 Unità Ginecologia Oncologica, Dipartimento Scienze Della Salute Della Donna E Del Bambino E Di Sanità Pubblica, Fondazione Policlinico Universitario A. Gemelli IRCCS, Largo Agostino Gemelli 8, 00168 Roma, Italy; 3 Dipartimento Diagnostica per Immagini Radioterapia Oncologica ed Ematologia, Fondazione Policlinico Universitario A. Gemelli IRCCS, Largo Agostino Gemelli 8, 00168 Roma, Italy; 4 Facoltà di Medicina e Chirurgia, Dipartimento di Medicina e Chirurgia Traslazionale, Università Cattolica del Sacro Cuore, Largo Francesco Vito 8, 00168 Roma, Italy

**Keywords:** vulvar reconstruction, vulvoperineal reconstruction, lichen sclerosus, vulvar stenosis, perforator flap, capillary perforator, introital stenosis

## Abstract

Background: Vulvar stenosis is a debilitating condition that compromises sexual function, urination, and the ability to undergo gynecological examinations. The purpose of this study is to describe the technique of capillary perforator perineal flaps (CPPF) for the correction of vulvar stenosis. Methods: We retrospectively examined patients with vulvar stenosis treated through surgical separation and reconstruction with CPPF. The procedure involved vulvar separation with the creation of a subsequent defect, repaired using a flap, harvested laterally to the labia majora including a capillary perforator and transferred through a subcutaneous tunnel to repair the vulvar defect. The functional outcome was evaluated with the Bradford scale, comparing the preoperative and postoperative scores using the Student’s *t*-test. Results: thirteen patients were included, three with stenosis following treatment for vulvar cancer and ten due to lichen sclerosus. In total, we analyzed 29 flaps, with an average size of 15.6 cm^2^. We always included just one perforator in the flap and no postoperative complications. Stenosis was resolved in all patients, with no recurrences one year after the surgery. The preoperative average severity of the stenosis was 2.3 + 0.6, reducing to 0.3 + 0.4 post-intervention, indicating a significant improvement (*p* < 0.01). Conclusions: CPPF has proven to be a quick and safe method for the reconstruction of vulvar stenosis.

## 1. Introduction

Vulvar stenosis is a debilitating clinical condition that significantly impacts patients’ quality of life and requires a specialized treatment approach. It is typically associated with congenital malformations, trauma, chronic inflammatory disease, vulvoperineal surgery, or radiation therapy [1]. A common cause of vulvar stenosis is scarring from previous surgery or lichen sclerosus (VLS), which leads to partial or total coverage of the vaginal introitus and urethral meatus, compromising sexual function, urinary flow, and the ability to undergo gynecological examinations [2,3]. Reconstructing this complex region is challenging, as it requires both functional and aesthetic restoration of an area that has been structurally altered by the underlying disease, where wound healing can be difficult [4,5]. Non-surgical treatments, such as topical creams and vaginal dilators, are largely ineffective in treating scar tissue constriction and the narrowing of the vaginal introitus, often necessitating a surgical approach. To our knowledge, there have been few reports describing the surgical repair of introital stenosis, with most using principles of plastic surgery involving techniques such as Z-plasty [6], vertical or horizontal tissue excision [7,8], split-thickness or full-thickness skin grafts [9], and local and regional flaps [10,11,12,13]. This study aims to describe the outcomes in a series of patients who underwent surgery for the correction of vulvar stenosis involving the vaginal introitus or the urethral meatus, and who were reconstructed using capillary perforator flaps harvested from the perineum, describing the surgical technique used.

## 2. Materials and Methods

We conducted a retrospective observational study examining the data obtained from clinical records, intraoperative findings, and follow-up visits. We recruited all patients with vulvar scars from previous surgery or lichen sclerosus that caused partial or total coverage of the vaginal introitus and urethral meatus who underwent contracture release and reconstruction using capillary perforator perineal flaps at our institution between January 2016 and November 2022. Patients who did not undergo follow-up at our center, or who had a follow-up of less than one year, or who had incomplete data were not included in the study. The variables of interest that we collected included age, height, weight, BMI, smoking habits, comorbidities, previous surgery, cause and duration of stenosis, complications, outcome of surgery, and months of follow-up. Prior to surgery, all patients underwent color Doppler ultrasound (CDU). We also took preoperative, intraoperative, and postoperative photographs. The procedure was performed by the senior author (S.G.) using a standardized technique that will be described below. The vulvar stenosis was categorized as “anterior” if the constriction was primarily present in the area above the urethral meatus, “posterior” if the stenosis predominantly involved the posterior commissure with a reduction of the vaginal introitus, and “complete” if both areas were affected. Follow-up visits were scheduled at 3 months and 1 year after the surgery. Two experienced plastic surgeons in gynecological reconstruction assessed the objective functional outcome during the clinical examination. The functional outcome was evaluated using a validated score to measure the severity of the introitus stenosis, according to the Bradford scale [14]: +1 indicates less than one third of the introitus obscured; +2 indicates one third to one half of the introitus obscured; and +3 indicates introitus admitting 1 examining finger only. We considered +0 to indicate normal introitus.

### 2.1. Statistical Methods

A detailed description of the sample’s clinical and demographic features was conducted using descriptive statistical techniques. Quantitative variables were described using various measures, including minimum, maximum, range, mean, and standard deviation. Changes between preoperative and postoperative introital stenosis degrees were analyzed using Student’s *t*-test, with *p* values < 0.05 considered significant. All statistical analyses were carried out using IBM^®^SPSS^®^Statistics software V.24 (IBM, Armonk, NY, USA).

### 2.2. Surgical Technique

The day before surgery, all patients underwent a CDU examination in the vulvoperineal region, using an 18–22 MHz transducer, in the lithotomy position. During the ultrasound examination, we located the internal pudendal artery and its two branches: the perineal artery and the clitoral artery. These arteries supply the labia and perineum through numerous terminal branches and capillary perforators. By following these vessels with CDU, it was possible to see their branches ascending into the subcutaneous tissue towards the skin as terminal branches or capillary perforators. The most superficial detectable point of these vessels, which essentially constitutes the entry point of the vessel into the skin, was marked with a dermatographic marker on the skin and included in the design of the flap.

The term “capillary perforators” was coined by Koshima [15] and refers to small perforators with a caliber generally ranging between 0.3 mm and 0.5 mm that rise vertically in the subcutaneous fat and vascularize the skin. The possibility of identifying the presence and studying the course of these vessels with CDU for flap preparation has already been reported by other authors [16,17].

We searched for capillary perforators in the area lateral to the labia majora. The flap design was sketched at this stage, positioning it at the level of the stenosis area, and the decision of whether to perform it unilaterally or bilaterally depended on the anticipated extent of the defect after scar removal. The flap was planned as a small rhomboid-shaped skin island, drawn between the labia majora and the groin crease, including the previously selected capillary perforator. The axis of the rhomboid was planned to be parallel to the groin crease. Obviously, this preoperative marking was reviewed during the procedure and potentially adjusted by modifying the outline or by performing the flap unilaterally if sufficient to successfully repair the defect. The surgery was performed under deep sedation, with a laryngeal mask, but without the need for significant muscle relaxation and in the lithotomy position with legs in stirrups. After preparing the sterile surgical field, a urinary catheter was placed, which was kept until the patient was discharged on the same day, or in the case of surgery performed in the late afternoon, on the following day.

We always began the procedure by excising retracting scars and correcting any potential bridging between the right and left sides of the vulva through manual lysis. Since these fusions are typically more extensive and functionally more relevant at the level of the commissures, we continued scar excision or release until achieving complete visualization of the vaginal introitus, urethral meatus, and, when possible, of the clitoris. Once this initial part of the procedure was completed, it resulted in defects of varying extent and position. For the repair of these defects, we verified the suitability of the preoperative design of the perineal flaps, potentially modifying it and deciding whether to perform the flap unilaterally or bilaterally. After a skin incision of the flap edges made with a cold blade, dissection of the subcutaneous fat was performed under magnification using 2.5× loupes, with a low-set electric scalpel and a blunt tip. The use of the electric scalpel was alternated with small bipolar microsurgery forceps. The subcutaneous tissue was carefully dissected all around the flap circumference, with a slightly slanted direction towards the vulvar defect. In most cases, it was possible to visualize the capillary perforator and follow its course toward the deep layers by microsurgical dissection.

In the absence of direct visualization of the vessel, no targeted search was performed, assuming that it was still located within the context of the dissected adipose tissue remaining attached to the skin island, on the basis of preoperative ultrasound localization. We have never performed complete skeletonization of the perforator to prevent vasospasm, which can be very dangerous given the delicacy of these small vessels. We never proceeded with the aim of precisely identifying the source vessel, effectively using a free style approach; as soon as we achieved sufficient mobility to transpose the flap into the defect without tension, we stopped the dissection. At this point, we created a subcutaneous tunnel beneath the major labium, wide enough to allow passage of the flap without compression. After completion of the flap transposition, the skin and subcutaneous tissue were sutured with 4-0 polylactic acid, achieving primary closure of the donor site and stabilization of the flap at the recipient site, repairing the defect. At the end, no vaginal stents were placed, and after surgery no patient was prescribed the use of postoperative splints or postural limitations.

## 3. Results

**Figure 1:**
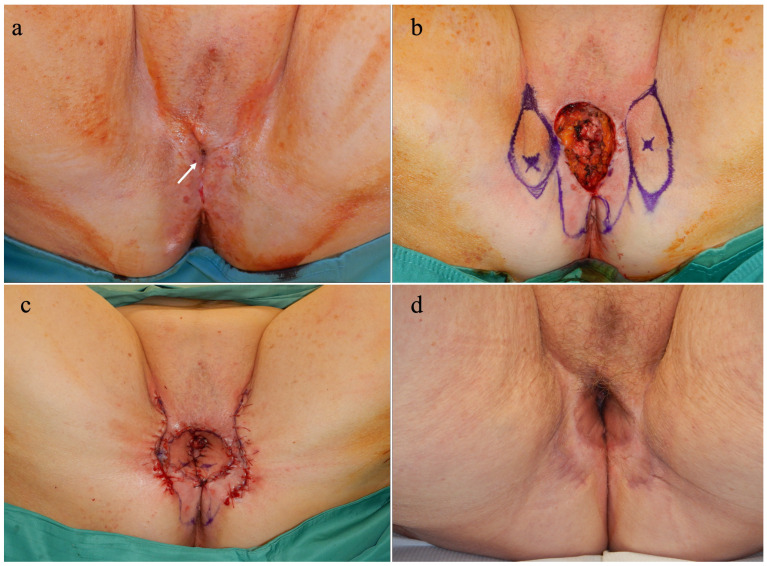
(**a**) This figure shows the case of an 85-year-old patient, with almost complete fusion of the vulva and obstruction of the urethral meatus and vaginal introitus. The remaining opening consists of only a small orifice, indicated by the white arrow. (**b**) The fusion was eliminated, exposing the vagina and urethra. Two perineal flaps were designed on two capillary perforators located by color Doppler ultrasound before the surgery and indicated by the small dot in blue. (**c**) The flaps were transposed to repair the defect, and the donor sites were repaired primarily. (**d**) The image of the patient one year after the surgery demonstrates the maintenance of a good opening of the vulva. The photos are original material by the authors.

**Figure 2:**
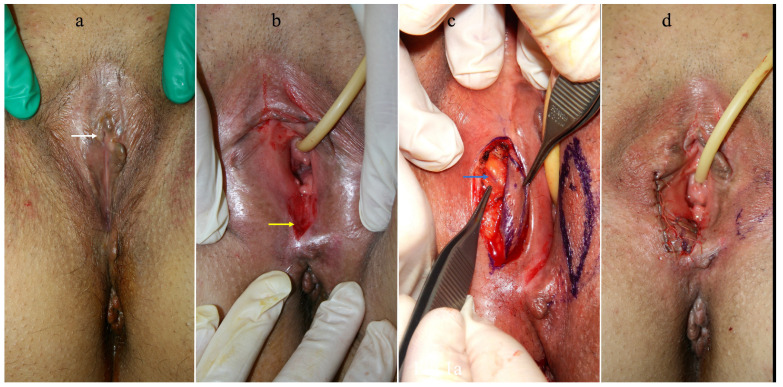
(**a**) This photo highlights a subtotal fusion of the vulva, resulting from lichen sclerosus, in a 62-year-old patient. The small remaining opening is indicated by the white arrow. (**b**) The appearance of the vulva after opening the stenosis. The full-thickness defect is localized exclusively at the level of the posterior commissure because the rest of the length of the vulva has been manually opened, creating only microscopic areas of abrasion in several points but not a real area of defect. (**c**) Only one capillary perforator perineal flap was harvested from the right side of the perineum. The perforator is clearly visible and indicated by the blue arrow. It is worth noting how the position of the perforator perfectly coincides with the blue point marked following the preoperative Doppler localization. (**d**) The flap transposed at the level of the posterior commissure, with stable restoration of the opening of the vaginal introitus. The photos are original material by the authors.

**Figure 3:**
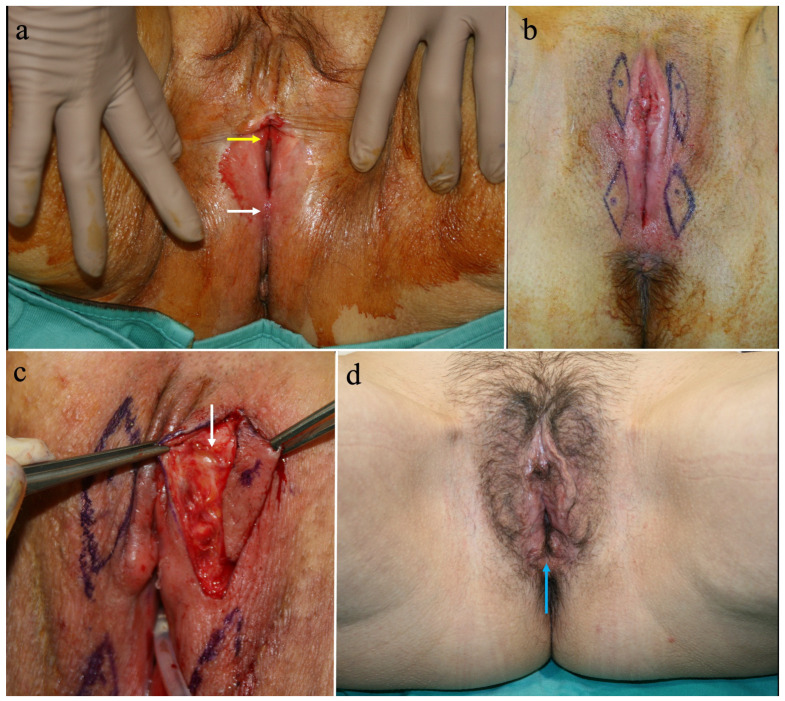
(**a**) The image of this 76-year-old patient shows an anterior vulvar fusion (yellow arrow) covering the urethral meatus and a posterior one (white arrow) reducing the vaginal introitus. (**b**) Four perineal flaps were marked based on four capillary perforators, detected by color Doppler examination and positioned at the level of the fusion areas, where the defects will likely be located after correcting the stenosis. (**c**) The capillary perforator of one of the flaps, highlighted by the white arrow. (**d**) One year after surgery, a normal vulvar opening was restored, with the presence of a normal vaginal introitus indicated by the blue arrow. The photos are original material by the authors.

Between January 2016 and November 2022, 13 patients underwent reconstruction with capillary perforator perineal flaps for the treatment of vulvar stenosis. In three patients, the cause of the stenosis was a previous ablative surgery for vulvar cancer, and in ten cases it was associated with VLS. The age of the patients ranged from 30 to 85 years, with an average of 59.5 + 13. BMI ranged from 16.18 to 31.56, with an average of 25.23 + 4.43. Regarding the type of stenosis, the examination of the patients revealed an anterior-only form in three cases, a posterior-only form in two cases, and complete involvement in eight cases. Complete vulvar involvement was observed in three patients with previous ablative surgery (Figure 1) and in five of those with VLS. A total of 29 flaps were used in the cohort of 13 patients: in 1 case unilateral (Figure 2), in 12 cases bilateral, and in 1 case 2 on each side (Figure 3).

It was always possible to identify a perforating vessel in the perineal area lateral to the labia majora preoperatively, at the level of the scar tissue to be removed or released. The dimensions of the flap ranged from 4 cm × 2.5 cm to 6 cm × 3.5 cm, with an average surface area of 15.6 cm^2^. The inclusion of a single perforator in the flap was sufficient to ensure good vascularization and we never observed any cases of necrosis, either partial or complete, or dehiscence. We did not observe any other complications in the postoperative period. The duration of the procedure ranged from 66 to 124 min, with an average of 96 min. All patients were able to achieve and maintain a normal urinary flow and the possibility of being examined by a gynecologist through direct inspection or using a speculum following surgery. During the follow-up, we did not detect any cases of stenosis recurrence. Before the intervention, the average value relative to the severity of the stenosis measured according to the Bradford scale was 2.3 + 0.6 and decreased to 0.3 + 0.5 after the intervention, indicating a significant improvement (*p* < 0.01) (Table 1).

## 4. Discussion

A thorough understanding of vulvovaginal anatomy and introital deformities is essential for the successful surgical repair of introital stenosis. Reconstructive techniques must be tailored to the individual case, considering factors such as the degree of stenosis, the anatomical subunits involved, and patient-specific characteristics like skin laxity and previous surgeries or radiotherapy [18,19]. Although various locoregional flaps are described in the literature for post-oncological vulvoperineal reconstruction [20,21,22,23,24,25,26,27], their use in managing vulvar stenosis is less documented. One case report by Buda et al. [12] reported the use of a tunneled modified lotus petal flap for vulvar reconstruction in a severe case of introital stenosis following radical vulvectomy. Other authors have employed rhomboid transposition flaps [8], pudendal–thigh flaps [13], and V-Y fascio-cutaneous advancement flaps [28] to address small vulvovaginal defects. However, these flaps often result in significant morbidity and scarring at the donor site, distorting surrounding structures [29,30,31]. Labia majora flaps, which adhere to the principle of reconstructing like-with-like, often yield better outcomes, as seen in reconstructions of the eyelids, lips, and nipples [10,11]. However, their use is not always possible, such as in cases of previous vulvectomy or the involvement of vulvar lichen sclerosus (VLS). Perineal flaps are a valid alternative, offering similar thickness and inconspicuous donor site scars. Several perforator flaps proposed for post-oncological vulvar reconstruction provide an ideal combination of thickness, texture, and flexibility compared to traditional flaps [32,33]. The classical perforator flap requires extensive dissection of the perforator and source vessel, which is time-consuming. In 2010, Koshima et al. [15] introduced the concept of capillary perforators—small vessels less than 0.5 mm in diameter. These were used to harvest a thoracodorsal artery perforator (TAP) flap for upper limb reconstruction, offering advantages such as easier elevation, larger flap size with stable perfusion, lower donor site morbidity, and the possibility of a thinner flap.

In our cases, the novelty of this reconstructive approach lies in using a flap with vascular safety ensured by a capillary perforator, while still maintaining a quick dissection that remains confined to superficial planes due to the short segment of the vessel needed for transposition. Microsurgical techniques, including the use of microsurgical forceps, bipolar forceps, and optical magnification with loupes, were employed for the dissection of these small perforators.

In 2015, Tashiro et al. [16,17] highlighted the utility of preoperative CDU examination to visualize capillary perforators, facilitating their successful inclusion in flaps. The perineum has a dense network of perforating blood vessels that originate from the obturator artery and the internal and external pudendal arteries and are interconnected by anastomoses and choke vessels. CDU is considered by many to be a useful and versatile tool for identifying and mapping the superficial subcutaneous structures in the female genital region, such as perforating vessels, lymphatic vessels, and lymph nodes [34,35,36,37,38,39,40]. In this study, CDU enabled the visualization and mapping of perineal capillary perforators, ensuring safer flap harvesting. Knowing the perforator’s position beforehand allowed for the flap position to be adapted to the patient’s needs. The perineal donor site is ideal, as it is usually outside disease-affected areas and has skin thickness similar to the vulva. Preserving subcutaneous fat around the perforator prevents lymphatic stasis, avoiding thickening or fibrosis post-surgery. Additional benefits include a low visibility of scars and quick patient recovery. However, a disadvantage of these flaps is the potential transfer of hair from the donor site to the reconstructed area. Older patients had sparse hair, which was not problematic, while younger patients with thicker hair underwent hair removal sessions or laser treatment. Capillary perforator flaps in the perineum are unsuitable for patients with previous radiotherapy [41] due to the high risk of damage to the perforators, especially venous ones, increasing the risk of complications. Our series consists of a small number of patients, which constitutes the main limitation of the study and makes an appropriate statistical analysis impossible. Additionally, further follow-up is necessary and is currently ongoing.

## 5. Conclusions

The introduction of capillary perforator perineal flaps offers significant improvements in surgical outcomes, ensuring vascular safety while maintaining a quick and superficial dissection process. Our results suggest that they are effective in resolving stenosis, safe, minimally invasive, and can be considered a valid option for functionally relevant vulvar stenosis. Overall, the evolution of these techniques and tools highlights the importance of personalized surgical approaches to optimize patient outcomes in vulvar stenosis management. Patients who should undergo these reconstructive procedures are those experiencing significant functional impairments, such as severe urinary issues or substantial difficulties with sexual intercourse and gynecological exams. Each case should be individually assessed, taking into account the degree of stenosis, anatomical considerations, and patient-specific factors. A larger number of patients is necessary to confirm our preliminary results.

## Figures and Tables

**Table 1:** Results and data of the patients.

Patients N = 13; flaps N = 29	
Age (mean, SD)	59.5 (13)
BMI (mean, SD)	25.2 (4.4)
Type of stenosis	
Anterior	3
Posterior	2
Complete	8
Type of diagnosis	
Lichen sclerosus	10
Previous surgery	3
Associated comorbidities	
Recurrent urinary infections	1
Hypertension	1
Hypothyroidism	1
Ischemic heart disease treated with previous aortocoronary bypass	1
Bradford Scale Score	
Preoperative (mean, SD)	2.3 (0.6)
Postoperative (mean, SD)	0.3 (0.5)
*p*-value	*p* < 0.01

## Data Availability

All relevant data are contained within the paper.
